# First Closed Genome Sequence of *Campylobacter fetus* subsp. *venerealis* bv. intermedius

**DOI:** 10.1128/genomeA.01246-13

**Published:** 2014-02-06

**Authors:** Linda van der Graaf-van Bloois, William G. Miller, Emma Yee, James L. Bono, Martine Rijnsburger, Carlos Campero, Jaap A. Wagenaar, Birgitta Duim

**Affiliations:** a Department of Infectious Diseases and Immunology, Faculty of Veterinary Medicine, Utrecht University, Utrecht, the Netherlands; b WHO Collaborating Center for *Campylobacter*/OIE Reference Laboratory for Campylobacteriosis; c Produce Safety and Microbiology Research Unit, Agricultural Research Service, U.S. Department of Agriculture, Albany, California, USA; d Meat Safety and Quality Research Unit, Agricultural Research Service, U.S. Department of Agriculture, Clay Center, Nebraska, USA; e VU University Medical Center, Department of Medical Microbiology and Infection Control, Amsterdam, the Netherlands; f Patología Veterinaria, Instituto Nacional de Tecnología Agropecuaria (INTA), Balcarce, Argentina; g Central Veterinary Institute of Wageningen UR, Lelystad, the Netherlands

## Abstract

*Campylobacter fetus* subsp. *venerealis* bv. intermedius is a variant of *C. fetus* subsp. *venerealis*, the causative agent of bovine genital campylobacteriosis, a venereal disease associated with abortion and infertility in cattle. We report the first closed whole-genome sequence of this biovar.

## GENOME ANNOUNCEMENT

The pathogen *Campylobacter fetus* includes two subspecies: *C. fetus* subsp. *fetus* and *C. fetus* subsp. *venerealis*. The niches of the subspecies are different: *C. fetus* subsp. *fetus* can be isolated from a variety of different hosts (1), whereas *Campylobacter fetus* subsp. *venerealis* is restricted to the genital tracts of cattle (2). Both subspecies can cause disease in cattle, but only *C. fetus* subsp. *venerealis* is described as the causative agent of bovine genital campylobacteriosis, a disease characterized by fertility problems in cattle (2). A variant of *C. fetus* subsp. *venerealis*, designated *C. fetus* subsp. *venerealis* bv. intermedius, has been identified (3). A *C. fetus* subsp. *venerealis* bv. intermedius genome sequence is available (4), consisting of 218 unassembled contigs. This unassembled genome does not allow for the full identification of the core and accessory genome and the reconstruction of pathways and surface structures that might contribute to the pathogenicity of this biovar. In this study, we report the first closed whole-genome sequence of *C. fetus* subsp. *venerealis* bv. intermedius strain 03/293, isolated in Argentina from the lung of an aborted bovine fetus (5).

For sequencing, three platforms were combined: Roche GS-FLX Titanium, Illumina MiSeq, and PacBio RS. A total of 248,123 Roche 454 reads were assembled using the Newbler assembler (version 2.6) into four scaffolds with 82 contigs, with 50× coverage. All 454 base calls were validated using 1,490,018 Illumina MiSeq reads, which added 156× coverage. A circular high-resolution AflII restriction map of the genome was generated by optical mapping (Argus Optical Mapper; OpGen, Inc., Gaithersburg, MD) to determine the orientation and order of the contigs and validate the assembly. The assembly of regions with insertion sequences or repeats in the S-layer and pathogenicity island (PAI) locus were confirmed with PacBio continuous long reads, adding 117× coverage. All base calls and polymeric tracts were validated using the high-depth MiSeq reads.

*C. fetus* subsp. *venerealis* bv. intermedius strain 03/293 has a circular genome of 1,866,009 bp, with an average G+C content of 33%. Protein-, rRNA-, and tRNA-encoding genes were identified as described previously (6). Annotation was based on *C. fetus* strains 82-40 (GenBank accession no. NC_008599) and 03-427^T^ (accession no. CP006833), using Artemis (7), and the identification of Pfam domains (version 27.0) (8). The genome contains 1,773 putative protein-encoding genes, 48 pseudogenes, 43 tRNA genes, and 3 rRNA operons. The genome also includes 13 insertion sequence elements and 31 homopolymeric GC tracts (≥8 bp). This strain is a carrier of two megaplasmids (91,400 bp and 35,326 bp) and a small cryptic plasmid (3,993 bp).

The *C. fetus* subsp. *venerealis* bv. intermedius genome contains an S-layer coding region, which is also found in *C. fetus* subsp. *fetus* and *C. fetus* subsp. *venerealis*. The genomes of the *C. fetus* subspecies were compared in a BLASTp analysis. High degrees of both synteny and similarity in gene content between the *C. fetus* subspecies were shown. The closed genome sequence of *C. fetus* subsp. *venerealis* bv. intermedius is an important reference genome for the comparison of virulence and host associations of the different *C. fetus* subspecies.

### Nucleotide sequence accession numbers.

The complete genome sequence of *C. fetus* subsp. *venerealis* bv. intermedius strain 03/293 has been deposited in GenBank under accession no. CP006999 to CP007002.
